# Copycat in Suicide: A Systematic Review of the Literature

**DOI:** 10.3390/jcm13237118

**Published:** 2024-11-25

**Authors:** Saverio Gualtieri, Maria Cristina Verrina, Matteo Antonio Sacco, Lucia Tarda, Luca Calanna, Jasmine Calafiore, Stefano Lombardo, Santo Gratteri, Isabella Aquila

**Affiliations:** Institute of Legal Medicine, Department of Medical and Surgical Sciences, University “Magna Graecia” of Catanzaro, Viale Europa, Loc. Germaneto, 88100 Catanzaro, Italy; saveriogualtieri@icloud.com (S.G.); mariacristina.verrina@studenti.unicz.it (M.C.V.); matteoantoniosacco@gmail.com (M.A.S.); lucia.tarda@studenti.unicz.it (L.T.); luca.calanna@studenti.unicz.it (L.C.); jasmine.calafiore@studenti.unicz.it (J.C.); stefano.lombardo@studenti.unicz.it (S.L.); gratteri@unicz.it (S.G.)

**Keywords:** copycat, suicide, homicide, murder

## Abstract

**Background**: The “copycat effect” is a psychological phenomenon in which a person’s actions influence the behavior of others, leading to imitations of behaviors, events, or ideas. It is often observed in contexts such as crimes, suicides, or violent behaviors, where media coverage of an event can trigger similar reactions in other people. Usually, many studies associate the copycat phenomenon with homicide or serial crimes. Little attention is paid to the phenomenon of emulation in suicide and, therefore, to the copycat phenomenon in this context. **Methods**: In our study, a systematic review of the literature was carried out using keywords related to copycat and suicide. Subsequently, each study was read and analyzed. **Results**: The results were compared with each other and demonstrated how it is essential to mitigate the resonance of suicidal events, especially through the media and social networks. In particular, this risk of emulation is greater among young people who are exposed to a continuous external source of information through news from the web in an incessant and constant way. This risk increases most immediately after the publicity of the suicidal event in a limited period of time. **Conclusions**: Unfortunately, little attention is given to the analysis of the copycat phenomenon, which causes an underestimation of the data related to suicide emulation, especially from the preventive perspective of a phenomenon that is still widespread.

## 1. Introduction

### 1.1. Understanding the Concept of Copycat Crimes

The Copycat model is a cognitive theory and computational framework that simulates how humans create analogies and find correspondences between concepts in a creative and flexible way. This model was developed to understand the cognitive processes that allow us to generate and apply analogies to solve complex problems, going beyond rigid rule-based systems. It is a tool to explore a fundamental aspect of human thought: the ability to identify similarities and relationships between different situations. The foundations of the Copycat model are based on specific concepts, such as the fluidity and adaptability of cognition, which operate in a dynamic and probabilistic “workspace”. A central element is the concept of “slippage”, the flexible remapping of relationships between elements in different contexts, enabling the creation of new correspondences. Additionally, the model works through emergent behaviors, where solutions and patterns arise from the interaction of low-level components, and it uses parallel scanning, simultaneously evaluating multiple possibilities, to reflect how human thought explores various creative paths. Created by Douglas Hofstadter and Melanie Mitchell, the Copycat model is not designed to directly understand an individual person but provides profound insights into the universal mechanisms of human analogical thinking. Copycat crimes are unique criminal behaviors characterized by their imitation of previous crimes, often sensationalized by media portrayals. These crimes occur when individuals replicate the actions of a notorious criminal or a high-profile criminal act, leading to similar offenses. The concept of copycat crimes is not limited to a specific type of criminal activity but can encompass a range of offenses, including theft, arson, and homicide. A defining characteristic of copycat crimes is their reliance on pre-existing criminal acts as templates, which individuals then emulate to achieve notoriety or fulfill some psychological need [1]. Data reveal that a significant portion of individuals involved in copycat murders already displayed a history of violence or had underlying mental instability before engaging in such crimes [2]. Psychological theories suggest that individuals with certain personality traits, such as narcissism or psychopathy, may be more inclined to commit copycat crimes. These individuals may perceive the act of copying notorious criminals as a way to construct their identity or to gain notoriety in a society that often glorifies such figures [2]. The impact of media coverage on the prevalence of copycat crimes is significant, as the media often serves as a catalyst for imitation. Extensive media coverage of violent crimes, especially those involving serial killers or mass murderers, can inadvertently glamorize these acts, making them appealing to certain individuals. Studies have shown that contemporary copycat crimes can be linked to the portrayal of violence in news media, films, and other forms of entertainment [3]. The sensationalism surrounding high-profile criminal cases can create a narrative that some find irresistibly compelling, leading them to replicate these acts in hopes of achieving similar attention.

### 1.2. Copycat and Epidemiology

Epidemiological studies have explored the spread of copycat phenomena, revealing patterns and consequences of media influence on behavior. Research indicates that media coverage can act as a catalyst for such behaviors, particularly in vulnerable populations [3]. For instance, the imitation of high-profile suicides or mass murders often follows intense media coverage, suggesting a correlation between the two [2]. These studies have utilized various methodologies, including analysis of death certificates and sociodemographic data, to understand the extent of the copycat effect [4]. Findings from these studies underscore the importance of responsible media reporting and the need for interventions to mitigate the impact of harmful media portrayals on public health. A recent study has shown that due to the heterogeneity of the studies published so far on this topic, it is still difficult to obtain data from an epidemiological point of view in terms of odds ratios, rate ratios, or standardized mortality ratios and also to conduct meta-analyses on this topic [5]. In North America and Europe, the phenomenon of copycat suicides has been extensively documented, indicating a significant increase in incidences following widely publicized suicides [6]. The impact is particularly pronounced among female and younger demographics, who appear more susceptible to imitating the suicidal behaviors of celebrities and public figures. In Asia and the Pacific regions, copycat suicides have emerged as a significant concern, particularly in countries like South Korea, where celebrity suicides have been closely linked to subsequent increases in suicide rates. For instance, the deaths of prominent figures such as Jonghyun, Sulli, and Hara Gu were associated with a substantial rise in suicide rates, with increases of 1.21, 1.30, and 1.28 times, respectively [7]. Another study from South Korea showed a clear increase of 16.4% in suicides in one day after the death of a celebrity, and this increase is greater in people who share the same characteristics as the celebrity [8]. The phenomenon has also been observed in other Asian countries, such as Hong Kong and India. Data from Africa and Latin America on copycat suicide occurrences reveal a somewhat different pattern, though the influence of media remains a critical factor [9]. While comprehensive studies are less prevalent in these regions compared to North America, Europe, and Asia, there is evidence to suggest that the Werther effect is not entirely absent.

### 1.3. Copycat and Homicide

The analysis of high-profile homicide cases reveals a significant influence on subsequent crimes, often referred to as the copycat effect. This phenomenon is particularly evident in cases involving mass shootings and serial killings, where the media coverage can inadvertently glamorize or sensationalize the perpetrators. This coverage can create a blueprint for individuals who might be prone to committing similar acts, seeing the attention given to such crimes as a form of validation or notoriety [10,11]. The impact is more pronounced in situations where the details of the crimes are extensively broadcasted, providing potential offenders with both inspiration and a method to execute their own crimes.

The risk of copycat mass killings with suicides is a significant concern in both public safety and mental health domains. This phenomenon, often termed the “contagion effect”, occurs when the extensive media coverage of a mass killing, particularly one involving a suicide, inspires subsequent similar acts by others. These events are typically high-profile and sensationalized, leading to the glorification or notoriety of the perpetrator, which can attract individuals already struggling with mental health issues, feelings of disenfranchisement, or desires for recognition. Mass killings involving suicides often share common characteristics, such as detailed planning, symbolic targeting, and the use of specific methods or locations. The perpetrators are frequently motivated by a combination of personal grievances, a desire for revenge, and a search for infamy. When such incidents are widely publicized, they can act as a blueprint for others who identify with the perpetrator or see the act as a way to express their own frustrations. Research indicates that the risk of copycat acts is particularly high when media coverage includes explicit details of the event, such as the method used, the personal background of the perpetrator, and their manifestos or motives.

### 1.4. Copycat and Serial Killer Murder

Research suggests that certain genetic factors, such as the presence of specific alleles, may predispose individuals to aggressive and violent behavior [12]. The serotonergic system, a part of the brain involved in regulating mood and aggression, has been identified as playing an important role in these predispositions. Genetic variants impacting this system could lead to heightened aggression and impulsivity, which are often observed in serial killers. One prominent example is the MAOA gene, often referred to as the “warrior gene”, which has been associated with increased aggression and violent tendencies [12]. Research has shown a significant correlation between certain MAOA genotypes and a disposition toward violence, suggesting that genetic makeup can be a determining factor in criminal behavior. These findings are bolstered by studies examining family histories, which often reveal patterns of violent behavior that can be traced back through generations, indicating a hereditary component [13]. The nature versus nurture debate in criminal psychology explores the complex interplay between genetic predispositions and environmental influences. While some argue that genetics lay the foundation for violent behavior, others emphasize the role of environmental factors like childhood trauma and social surroundings. Literature suggests that both elements are likely intertwined, with genetics providing a predisposition that environmental factors can either exacerbate or mitigate [13].

Serial killers often leave behind specific patterns or signatures, which are sometimes replicated by copycat offenders. These patterns may include the method of murder, choice of victim, or even the location of the crime. Copycat killers are known to meticulously study the behaviors and techniques of infamous serial killers, seeking to emulate their notorious crimes. This replication can provide the copycat with a sense of power and identity, often leading them to seek fame by committing more heinous acts than their predecessors [1].

### 1.5. Copycat and Suicide

Media coverage significantly impacts suicide rates, often contributing to a phenomenon known as the Werther effect. This effect is named after Goethe’s novel “*The Sorrows of Young Werther*”, which led to a spate of suicides among young men imitating the protagonist. When media reports on suicide events, particularly those involving celebrities, without discretion, it can inadvertently lead to an increase in suicide rates among vulnerable individuals [4]. The media’s portrayal of these events can glamorize or sensationalize the act, making it appear as a viable solution to personal struggles. Furthermore, detailed descriptions of the suicide method and the idolization of the deceased can exacerbate this effect, leading to a ripple of imitation among susceptible populations. Consequently, the media’s role becomes crucial in shaping public perception and potentially influencing suicidal behavior.

Certain populations are more susceptible to imitating suicidal behavior, highlighting the need for targeted preventive measures. Vulnerable groups often include individuals experiencing mental health issues, such as depression or anxiety, who may be seeking validation or a sense of belonging [14]. Adolescents and young adults are especially at risk due to their impressionable nature and the developmental stage of their cognitive and emotional faculties. Additionally, individuals exposed to similar life stressors or who identify closely with the deceased may feel an intensified connection, prompting imitative behavior. The presence of suicidal ideation or a history of previous suicide attempts further amplifies the risk within these populations.

### 1.6. Psychological and Emotional Factors

The role of mental health issues in suicide emulation is both significant and multifaceted. Mental disorders, including depression, anxiety, and psychotic disorders, are major contributors to suicidal behavior. These conditions often lead to a state of despair and hopelessness, which can compel individuals to emulate suicidal acts they have been exposed to, whether through media or personal connections. The presence of mental health issues can increase vulnerability to suicidal ideation and attempts, as individuals may perceive suicide as a viable escape from their emotional turmoil [15]. The risk is compounded when there is a history of mental illness or previous suicide attempts in the family, as this can normalize the act of suicide and lower the psychological barriers to emulating such behavior [16]. Addressing mental health issues through early intervention and treatment is crucial in breaking this cycle of emulation and preventing suicide [17].

Emotional distress plays a critical role in influencing the choice of suicide method among individuals prone to emulation. Those experiencing intense emotional pain may gravitate towards methods they perceive as quick and definitive, often influenced by the methods used in prior suicides they are aware of. For instance, drug poisoning might be chosen by attempters who seek a method that allows for contemplation and potential rescue, while more lethal methods like hanging are favored by completers who desire certainty. The emotional state of the individual, characterized by feelings of desperation and urgency, can dictate the method selected, further underscoring the importance of emotional regulation and support systems in suicide prevention. By understanding the emotional triggers that guide method selection, interventions can be tailored to address the underlying distress and reduce the propensity for emulation.

Personality traits significantly impact susceptibility to suicide emulation, with certain characteristics predisposing individuals to higher risks. Traits such as impulsivity and neuroticism are closely linked to suicidal behavior, as they can lead to hasty decisions without full consideration of the consequences. Young adults, in particular, exhibit a strong connection between personality characteristics and suicidal ideation, which tends to diminish with age [18]. This suggests that interventions targeting personality traits may be most effective when implemented early in life. Moreover, hopelessness and extroversion have been identified as promising indicators for risk screening across various suicidal behaviors, highlighting the need for personalized approaches in prevention strategies [19]. By recognizing and addressing these personality factors, it is possible to reduce the likelihood of suicide emulation and foster resilience against such tendencies.

### 1.7. Copycat and Social Media

Social media platforms play a crucial role in facilitating the spread of copycat behaviors, significantly impacting public behavior patterns. These platforms provide a vast and instantaneous means of communication, allowing information and trends to spread rapidly across millions of users worldwide. The anonymity and reach of social media enable individuals to share and amplify behaviors, sometimes with harmful consequences. This is particularly evident in the context of violent acts and suicidal behaviors, where social media can serve as both a catalyst and a medium for imitation. For example, mass shooters often seek notoriety and emulate previous incidents, using social media as a tool to broadcast their intentions and gain attention [1]. This dynamic relationship between social media and copycat behaviors underscores the importance of scrutinizing the content shared on these platforms and understanding its potential ramifications.

Case studies of viral challenges and their real-world consequences highlight the tangible effects of social media-fueled copycat behaviors. Viral challenges, often designed to be entertaining or daring, can sometimes lead to dangerous outcomes when individuals attempt to replicate them without considering the risks involved. A notable instance is the Tide Pod Challenge, where participants ingested laundry detergent pods, leading to severe health issues and hospitalizations. Similarly, the Blue Whale Challenge, which reportedly encouraged self-harm and suicidal acts, underscores the dire consequences of such viral phenomena. These cases demonstrate how social media can transform seemingly harmless trends into hazardous behaviors, emphasizing the need for greater awareness and intervention. The widespread nature of these challenges and their potential for harm necessitates a proactive approach to monitoring and mitigating their impact on public safety.

As demonstrated, copycat is a known phenomenon in serial crimes and murders. In the context of suicides, however, few studies have demonstrated a real correlation. The aim of this study is to refine the understanding of the state of the art in suicides where the copycat phenomenon can be detected, to analyze the characteristics of this phenomenon, and to test the following hypotheses: the null hypothesis (H0), that no significant association exists between the copycat phenomenon and an increase in suicide/homicide rates, and the alternative hypothesis (H1), that the copycat phenomenon is associated with an increase in suicide/homicide rates and presents specific identifiable characteristics.

## 2. Materials and Methods

**Figure 1:**
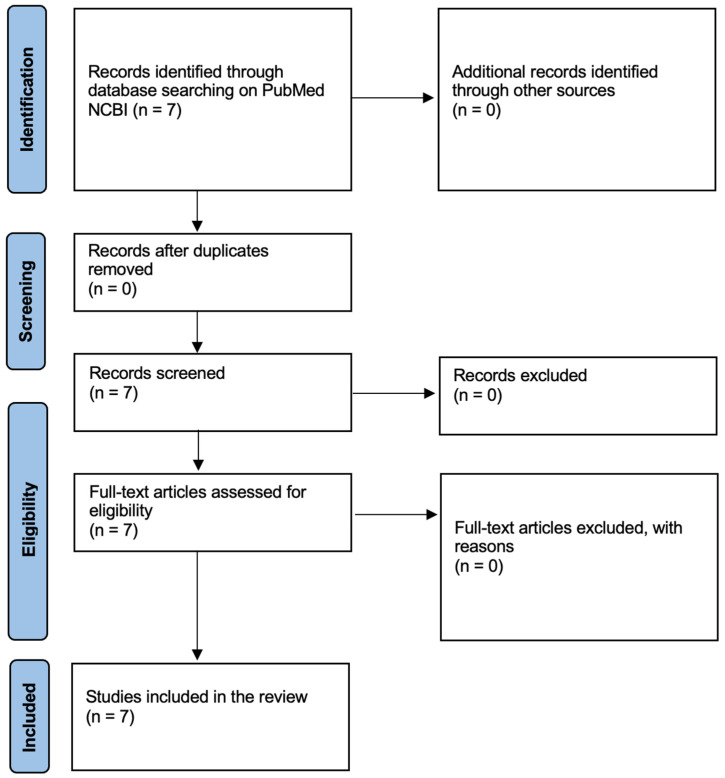
PRISMA flowchart.

A systematic review of the literature was performed through the Pubmed NCBI search engine, using the keywords “copycat and suicide and homicide and murder”. Inclusion criteria included works that analyzed the impact of the copycat phenomenon on suicide or homicide. Exclusion criteria included the removal of papers that did not investigate the correlation of the copycat with suicide or homicide. The works were selected after an initial reading of the abstract and title. Further, full texts were analyzed. Only epidemiological and forensic works were included in the review. All other works were excluded from the search. This review followed the PRISMA guidelines but was not registered (Supplementary Materials) (Figure 1).

## 3. Results

The literature analysis highlighted seven scientific works using the above-mentioned keywords. Among these seven works, three studies concerned the role of social media in the copycat phenomenon in suicides [9,20,21]. Vuorio et al. instead demonstrated an association between the increase in pilot aircraft-assisted suicides and the copycat phenomenon after terrorist attacks (such as 11 September 2001) [22]. Lankford et al. hypothesized a correlation between mass killings (including suicides) and subsequent suicidal phenomena, especially in the time frame represented by 14 days after the event [23]. A study by Lindberg et al. demonstrated an association between the copycat effect and suicidal/homicidal plans among Finnish adolescents with mental disorders [24]. Only one case concerned a death due to suicide by suffocation in a plastic bag related to the copycat effect [25] (Table 1).

From the literature review carried out, not many works emerged regarding the association of copycats with suicide, and this, therefore, demonstrates how the study of this phenomenon is underestimated.

## 4. Discussion

### 4.1. The Complexity of Copycat Phenomenon

Suicide emulation, or copycat suicide, involves an individual attempting suicide after being influenced by another’s suicide, often replicating the method or circumstances. This behavior is amplified by media coverage, which may sensationalize or romanticize the act, making it more appealing to vulnerable individuals. Recognizing the impact of social and media influences is key to preventing further incidents. Historically, suicide emulation has been documented across cultures, such as the “Werther effect”, where Goethe’s novel *The Sorrows of Young Werther* led to a series of suicides among young men. Psychological factors, such as mental health disorders like depression or anxiety, increase susceptibility to emulation, especially when individuals view suicide as a solution to their distress. Personality traits like impulsivity or imitation tendencies further predispose individuals to engage in copycat suicides [15,16,17,18,19,20,21,22,23,24]. Social isolation and poor coping mechanisms also play a role. Media portrayals, especially detailed depictions of methods, can encourage emulation, with the “Werther effect” showing that sensationalized reports of suicide methods influence vulnerable individuals. Social media, with its rapid dissemination of harmful content, further exacerbates this trend, especially among younger populations. Cultural attitudes towards suicide shape how it is perceived and the methods chosen, with some societies stigmatizing it and others seeing it as a solution to hardship. For example, Kurt Cobain’s suicide led to a rise in similar suicides among young people, highlighting the media’s role in shaping perceptions and triggering suicidal behavior. Increased exposure to suicide stories, especially on online platforms, has been linked to an increase in suicidal thoughts, further showing the cyclical nature of suicide emulation [17]. Gender differences in suicide emulation show that males have a higher rate of completed suicides, often using more lethal methods like hanging, while females are more likely to attempt suicide with less lethal methods, such as drug overdoses [19]. This difference suggests men have higher suicide intent, opting for irreversible methods [21]. Women, however, are less likely to complete suicide due to the use of non-lethal means. These patterns emphasize the need for gender-specific prevention strategies [15,16,17,18,19,20,21,22,23,24,25,26,27,28,29,30,31,32,33,34,35,36,37,38,39,40]. Addressing these dynamics with gender-specific prevention strategies is crucial for reducing suicide rates and improving mental health outcomes [15,16,17,18,19,20,21,22,23,24].

### 4.2. Analysis of Results in Context of Other Evidence

This review underscores the pervasive influence of media and societal factors on the emulation of suicides, often referred to as the “copycat” phenomenon. In the literature review, much attention is paid to the epidemiological data on the influence of the media on the copycat phenomenon. Quarshie correlated the ways in which Ghanaian online media report suicides with the risk of the copycat effect [9]. Auxemery examined the history of mass murderers, also analyzing the suicidal dimension that characterizes them, highlighting how both murders and suicides are often imitated and emphasizing the crucial role the media can play in preventing these events by adhering to WHO recommendations [20]. Machlin et al. described the characteristics of suicidal acts reported by the media and highlighted how suicides disseminated by the mass media can be subject to the copycat phenomenon [21].

Furthermore, one author focused on the temporal analysis of the genesis of copycat events, identifying a time frame that can be defined as “at risk” for emulation leading to subsequent suicidal acts [23]. This demonstrates how both the quantity and quality of information conveyed—particularly in a short time frame—can generate a phenomenon of emulation, not only in homicide crimes but especially in suicide. This emulation can involve the method chosen, the circumstances under which it occurred, conceptual plans for implementation, and the involvement of tools or means necessary to execute the plan and replicate the specific chronology of events desired by the individual.

The review of the literature underscores how little attention this phenomenon has received from the forensic scientific community and public health sector. Furthermore, it is a poorly studied phenomenon, and consequently, the data presented in the literature on this topic are significantly underestimated. The data that emerged show that the copycat phenomenon is almost always influenced by the media [8,20].

In our systematic review of the literature, we analyzed a particular case in which Perdekamp et al. described a suicide due to suffocation with a plastic bag, emulating a scene seen in a movie. This review also included a case of homicide carried out using the same method depicted in the film Charade [25]. From the analysis, it emerged that Yann Auxemery et al. conducted a narrative review emphasizing the role of the mass media in both mass murders and suicides [20]. For this reason, the WHO has stressed the need for accurate and responsible reporting by journalists. The analysis of the studies shows how underestimating the copycat phenomenon negatively affects the prevention of suicidal acts. The literature clearly indicates that mass media and social networks should be used with caution in distributing and describing news to minimize the adverse outcomes of copycat behavior. From a sociological perspective, especially in the post-COVID-19 era, younger age groups—particularly those under 18—who have greater exposure to social media and the virtual world are at increased risk. This risk is heightened when such events are reported and described repeatedly and prominently as front-page news on various social media platforms.

The findings align with previous evidence documenting the “Werther effect”, where sensationalized reporting or fictional depictions of suicide can lead to imitation, especially among vulnerable individuals. Historical cases, such as those linked to Goethe’s *The Sorrows of Young Werther*, and contemporary analyses highlight the media’s role in amplifying these risks. Media portrayals that include explicit descriptions of suicide methods, motives, or identities of individuals who die by suicide are particularly problematic, as they can inadvertently glamorize or normalize the act. Furthermore, emerging digital platforms, such as social media, have created new pathways for the rapid dissemination of harmful content, compounding the risks.

Notably, there is some variation in the impact of media coverage across regions and populations. For example, while the copycat effect is most pronounced in Western countries, it is somewhat less documented in parts of Asia and Latin America. Studies from South Korea showed a substantial increase in suicides following celebrity suicides (1.28 times the usual rate after the death of Hara Gu) [7], whereas data from India indicated a much lower correlation between media coverage of suicides and copycat incidents. These regional differences underscore the importance of considering cultural and social contexts in the interpretation of the copycat effect.

The review also highlights the intersection of psychological, sociocultural, and demographic factors in shaping suicidal behavior. Vulnerable populations, particularly adolescents and individuals with pre-existing mental health conditions, are disproportionately affected by exposure to suicide-related content. Additionally, gendered patterns of suicide emulation are evident, with males more likely to emulate highly lethal methods and females exhibiting preferences for less lethal options. These disparities point to the need for tailored interventions that address specific demographic and cultural contexts [15,16,17,18,19,20,21,22,23,24].

### 4.3. Limitations of the Evidence

The evidence base for suicide emulation is limited by several methodological constraints. Much of the available research relies on observational and correlational designs. Studies frequently depend on retrospective analyses or media content reviews, which can introduce bias and limit generalizability. The heterogeneity of methodologies, including varied definitions of “copycat” behavior and inconsistent metrics for assessing media influence, further complicates the synthesis of findings. Moreover, the temporal nature of emulation, with heightened risks immediately following media exposure, is often underexplored in long-term studies.

From a sociocultural perspective, there is limited understanding of how regional, cultural, and economic differences modulate the copycat phenomenon. For example, variations in cultural attitudes toward suicide or differences in media regulations likely influence outcomes, but these factors are underrepresented in the literature. Additionally, while digital media’s role in suicide contagion is recognized, many studies fail to capture the unique dynamics of social media platforms, such as algorithm-driven amplification of content.

This review has its own limitations, including the low number of studies identified on this topic. Furthermore, most of the studies have presented qualitative results rather than meta-analytic approaches, limiting the ability to quantify effect sizes. Emerging research on the influence of social media and other modern communication channels remains sparse, leading to gaps in understanding how contemporary media practices influence the copycat phenomenon in different parts of the world.

### 4.4. Implications for Practice, Policy, and Research

The findings of this review have significant implications for practice, policy, and research. Responsible media reporting should be prioritized by adopting and enforcing journalistic guidelines, such as those outlined by the WHO. These guidelines aim to minimize the risk of suicide contagion by avoiding sensationalism, refraining from describing methods in detail, and including mental health resources in coverage. Adhering to these recommendations has been shown to reduce copycat suicides in various regions, with one study in Australia showing a 20% decrease in suicide rates among young adults following their implementation [5]. Alongside the WHO’s guidelines, media literacy programs are also crucial in reducing the spread of harmful behaviors. These programs aim to educate the public, particularly vulnerable populations, about the negative effects of consuming sensationalized media, with research from the UK indicating a 15–25% reduction in imitation of violent crimes among adolescents [9]. Furthermore, collaborative efforts between law enforcement and media outlets have proven effective, as demonstrated in Sweden, where such collaborations led to a 30% reduction in the imitation of high-profile violent crimes. By referencing these evidence-based strategies, we can offer a more robust and actionable framework for practitioners and policymakers to address the risks associated with media-induced copycat behaviors. Stricter social media content moderation policies are also necessary to limit the dissemination of harmful material. Algorithms that prioritize sensational content should be redesigned to promote responsible reporting. Public awareness campaigns are essential to educate the general public, particularly youth, about the risks of sensationalized media and the importance of seeking help during crises. Future research should prioritize longitudinal studies to explore causal relationships between media exposure and suicide emulation. Cultural and regional analyses are needed to understand how norms and regulatory environments affect the copycat phenomenon. Investigating social media dynamics, such as content virality and user engagement, is critical to mitigating modern suicide contagion risks. Additionally, research should focus on demographic-specific prevention strategies for at-risk groups, including adolescents, men, and individuals with pre-existing mental health conditions.

### 4.5. Preventive Measures and Strategies

Intervention programs play a crucial role in reducing the emulation of suicide by providing targeted support and resources to individuals at risk. Effective programs often incorporate a combination of therapeutic interventions, education, and community support, addressing the root causes of suicidal behavior and mitigating the risk of contagion. For instance, meta-analyses have shown that antidepressants can significantly prevent suicide attempts, although individual randomized controlled trials may lack sufficient power to confirm these findings conclusively [26]. Intervention programs that integrate evidence-based treatments, such as cognitive–behavioral therapy and dialectical behavior therapy, are instrumental in preventing suicidal behavior and reducing the likelihood of imitation among vulnerable populations [29]. By offering comprehensive care and promoting resilience, these programs contribute to breaking the cycle of suicide emulation and fostering long-term recovery.

The importance of responsible media reporting on suicides cannot be overstated, as the way suicidal behavior is portrayed can significantly impact public perception and behavior. Research indicates that media reporting and portrayal of suicidal acts can have negative influences, potentially facilitating suicidal behavior among those exposed to such information [30]. Studies have also shown that media items about suicide can be associated with increases in actual suicide rates, highlighting the critical need for careful and sensitive reporting [16]. Responsible reporting practices, such as avoiding sensationalism and providing information on where to seek help, can encourage open conversations about suicidality and stimulate help-seeking behavior among individuals considering suicide [31]. By adhering to ethical guidelines, media outlets can play a pivotal role in preventing suicide emulation and promoting a more informed and supportive public discourse.

Strategies for promoting mental health awareness and support are essential in preventing suicide emulation and fostering a supportive environment for those at risk. A system-level approach that combines education, training, and screening has shown promise in reducing suicide risk by addressing mental health challenges at multiple levels [32]. E-health tools, which include education and awareness, self-screening, and access to support resources, are emerging as valuable components of suicide prevention strategies [33,34]. These tools empower individuals to take charge of their mental well-being and seek help when needed. Additionally, community-based programs that focus on promoting mental health and preventing substance abuse, such as those targeting AI/AN young people, are critical in building resilience and reducing the incidence of suicide. By prioritizing mental health education and support, society can create a more informed and compassionate environment that discourages suicide emulation and supports recovery.

## 5. Conclusions

The phenomenon of suicide emulation, driven by media and societal factors, presents a significant public health challenge. The findings emphasize the need for responsible media practices, stricter regulation of digital platforms, and comprehensive public health interventions. By advancing research and implementing evidence-based strategies, it is possible to mitigate the risks associated with the copycat phenomenon and foster a safer environment for vulnerable individuals. Collaboration among policymakers, mental health professionals, media outlets, and community organizations will be essential in addressing this complex and multifaceted issue. To this end, we propose that such news be filtered by the journalistic industry and social media, avoiding excessive description of the facts or emphasis on the narration of the smallest details of the chosen suicide methods and the dynamics related to them.

## Figures and Tables

**Table 1:** Results of the literature review.

Authors and Year	Type of Paper	Type of Journal	Results
Quarshie et al., 2021 [9]	Original article	Public health	The online reportage in Ghana of suicidal behavior does not reflect the WHO guidelines.
Vuorio et al., 2018 [22]	Original article	Public health	The risk of copycat aircraft accidents due to pilot suicide increased after the first year after 11 September 2001
Lankford et al., 2018 [23]	Original article	Psychiatry	Mass killings with suicides show an increased risk during the first 14 days
Auxemery et al., 2015 [20]	Narrative review	Psychiatry	Mass media play a crucial role in copycat tragedies
Machlin et al., 2013 [21]	Original article	Public health	Mass media can heighten the risk of imitative behaviors
Lindberg et al., 2012 [24]	Original article	Psychiatry	Copycats are associated with psychiatric symptoms such as anxiety and depression
Perdekamp et al., 2001 [25]	Case report	Psychiatry	A copycat suicide was performed simulating a movie scene

## Data Availability

Not applicable to this article as no datasets were generated.

## Supplementary Materials

The following supporting information can be downloaded at: https://www.mdpi.com/article/10.3390/jcm13237118/s1, Table S1: PRISMA checklist.
